# Electrophysiological findings during re-do procedures after single-shot pulmonary vein isolation for atrial fibrillation with pulsed field ablation

**DOI:** 10.1007/s10840-023-01559-z

**Published:** 2023-05-17

**Authors:** Federico Tancredi Magni, Daniel Scherr, Martin Manninger, Christian Sohns, Philip Sommer, Tatevik Hovakimyan, Yuri Blaauw, Bart A. Mulder

**Affiliations:** 1 Department of Cardiology, University of Groningen, University Medical Center Groningen, P.O. Box 30.001, 9700 RB Groningen, The Netherlands; 2 Department of Medicine, Division of Cardiology, Medical University of Graz, Graz, Austria; 3 Clinic for Electrophysiology, Herz- und Diabeteszentrum NRW, Bad Oeynhausen, Germany; 4 Department of Cardiac Arrhythmology, Nork-Marash Medical Center, Armenak Armenakyan 108/4, Yerevan, Armenia

**Keywords:** Atrial fibrillation, Pulmonary vein isolation, Pulsed field ablation, Repeat ablation

## Abstract

**Background:**

Pulsed field ablation (PFA) is a novel ablation technology recently adopted in the treatment of atrial fibrillation (AF). Currently, little is known about the durability of PFA ablation lesions.

**Methods:**

We investigated patients who underwent redo-ablation due to recurrent AF/atrial-flutter or tachycardia (AFL/AT) following PVI with PFA. We report electrophysiological findings and ablation strategy during redo-ablation.

**Results:**

Of 447 patients undergoing index PVI with PFA, 14 patients (age: 61.9±10.8 years; 7 (50.0%) males; left atrial volume index (*n*=10): 39.4±14.6 mL/m^2^) were referred for redo-ablation. Initial indication was paroxysmal-AF in 7 patients, persistent-AF in 6 and long-standing-persistent-AF in one patient. Mean time-to-recurrence was 4.9±1.9 months. Three patients received additional posterior-wall-isolation during index PFA. Twelve (85.7%) patients suffered AF recurrence and 5/12 had concomitant AFL. In the remaining 2 patients, one had a (box-dependent) AFL, and one had an atypical AT. No patients had all PVs reconnected. Reconnection in zero, one, two or three PVs was found in 35.7%, 21.4%, 14.3%, and 28.6% of patients, respectively. All 7 patients with zero or one reconnection with AF recurrence received additional/repeat posterior-wall-isolation during re-ablation, while in the others, PVs were re-isolated. Patients with only AFL/AT had no reconnection of PVs, and the substrate was successfully ablated.

**Conclusions:**

Durable PVI (all PV’s isolated) was observed in over one-third of patients at re-do. The predominant recurrent arrhythmia following PVI-only was AF. Concomitant (35.7%) or isolated (14.3%) AFL/AT recurrence was observed in 50% of patients.

## Introduction

Atrial fibrillation (AF) is the most common sustained arrhythmia conferring substantial burden of cardiovascular morbidity, mortality and impaired quality of life [1]. Pulmonary vein isolation (PVI) has become the mainstay of AF management if a rhythm control strategy is chosen [1, 2].

Pulsed field ablation (PFA) is a novel non-thermal ablation modality which applies electroporation to destabilize the cell membranes and form nanoscale pores, eventually resulting in irreversible damage and cell death [2–4]. Human tissues differ by their affinity to dielectric cell membrane breakdown. Myocardium has a lower threshold for tissue necrosis and this allows to preferentially ablate myocardial tissue without compromising surrounding anatomical structures such as oesophagus, blood vessels or nerve fibres [5]. PFA’s unique ability of selective electroporation and potential for minimal adnexal injury opens new, promising horizons into catheter ablation of AF [6]. However, one of the major issues to address is long-term results of the technique [4, 7]. In addition, questions remain regarding recurrent atrial flutter/tachycardia (AFL/AT) due to potential iatrogenic atrial channels following single-shot PFA.

In the present study, we present a series of 14 patients in whom a recurrence of AT/AFL/AF was observed within the first year after PVI with PFA. We provide an overview with patient characteristics and procedural findings of our first redo procedure for an atrial arrhythmia after an index procedure with PFA.

## Methods

### Patient population

In this study, we investigated 14 patients who underwent a re-ablation procedure within one year after being treated for symptomatic AF using PFA between April 2022 and October 2022 at the University Medical Center Groningen (UMCG), the Netherlands, the University Hospital Graz, Austria, and Clinic for Electrophysiology, Herz- und Diabeteszentrum NRW, Germany. Patients with paroxysmal as well as persistent AF were considered eligible for the procedure [1]. Anticoagulation was initiated at least 4 weeks before the procedure and continued for at least 3 months afterwards.

### PFA procedure

All patients underwent PFA under deep sedation using propofol and fentanyl. After transseptal puncture, a 13-F steerable sheath (Faradrive, Farapulse Inc.) was advanced into the left atrium via a guide wire. Then, a 12-F over-the-wire pentaspline PFA catheter (Farawave 31 or 35 mm, Farapulse Inc.) was introduced into the left atrium via the sheath. Heparin (70-100 E/kg) was given prior to transseptal puncture, and the target ACT was > 300 s. All pulmonary veins (PVs) were canulated with a J-wire guidewire (Amplatz extra stiff; Cook Inc. or Merit InQwire, .035”) and a total of eight PFA lesions were applied per vein, 4 in ‘basket’ and 4 in ‘flower’ configurations with a pulsed field amplitude of 2.0 kV. The application protocol for the left common ostium (LCO) was dependent on the size of the LCO. For LCOs larger than the catheter’s diameter in the basket position, applications in the basket configuration were delivered in separate veins, and applications in the flower positions were delivered ideally at the LCO itself. If the LCO was narrower than the catheter’s diameter in the basket position, then applications in both basket and flower position were delivered at the LCO.

In a subgroup of persistent AF patients, left atrial posterior wall isolation (PWI) was performed. The way the PWI was performed was left to the operator discretion (i.e. number of applications and sequence). For these procedures, 3D electroanatomic mapping was used (EnSite Precision™, Abbott, or Rhythmia HDx™, Boston Scientific, Marlborough, Massachusetts) and the 3D anatomy was created with a high-density mapping catheter (Advisor™ HD grid, Abbott, or Intellamap Orion™, Boston Scientific) or with the Farawave (Farapulse) catheter. After isolation of every PV the catheter in the flower shape — with the guidewire still in the PV ostium — was positioned against the posterior wall and two applications were delivered. This process was repeated for each PV. Subsequently, overlapping applications across the entire posterior wall were performed to ensure redundant coverage of the entire posterior wall. Before each application, the position of the Farawave® catheter was depicted on the 3D map using a ‘shadow’.(6,8) Following ablation, remapping was performed to verify the presence of a posterior box lesion. Pacing was performed for exit block. No oesophageal temperature probe was used.

If applicable, synchronised cardioversion to sinus rhythm was performed, and all entrance- and exit-block of all veins were confirmed with the PFA catheter expanded in ostial positions of the PV. After the procedure, patients were followed up at the operator’s discretion. In case of documented AF recurrences after a blanking period of three months, patients were offered a re-do-ablation.

### Repeat procedure

Repeat procedures were performed under deep sedation using propofol and fentanyl. 3D electroanatomic mapping systems were used for all repeat procedures (CARTO 7, Biosense Webster or Ensite Precision Mapping System, Abbott). After transseptal puncture, a multipolar mapping catheter (PentaRay, Biosense Webster or HD grid, Abbott) was used to map the left atrium. In case of AF, cardioversion was performed before mapping and in case of atypical flutter, activation mapping was performed. If reconnection of the PV was identified, radiofrequency ablation (Thermocool SmartTouch or QDOT Micro, Biosense Webster or Tacticath SE, Abbott) was performed at the reconnection site to re-isolate the vein, which was confirmed by entrance-exit block pacing. Atypical flutter ablation, rendering the arrhythmia non-inducible, was performed at the operator’s discretion using linear ablations. Ablation of the cavotricuspid isthmus with the endpoint of bidirectional block was performed in case typical atrial flutter was documented either before or during the procedure.

### Outcomes

The primary outcome was identification of rate and site of PV reconduction during redo ablation. Secondary outcomes included description of recurrent arrhythmias and of the ablation strategy employed to terminate it.

### Periprocedural outcomes

Procedural findings during index and redo ablation are described including procedure time (skin-to-skin: from venous puncture to sheath removal), confirmed isolation of the PVs, and number of applications per PV, as well as procedure-related complications.

### Statistical analysis

Patient characteristics, rate of complications, and procedure-related data are presented as mean and standard deviation or median and interquartile range for continuous and categorical variables, respectively. The analyses were conducted using IBM SPSS Statistics for Windows, version 23 (IBM Corp., Armonk, New York) and statistical significance was set at a p-value smaller than 0.05.

## Results

### Patient characteristics

**Fig. 1:**
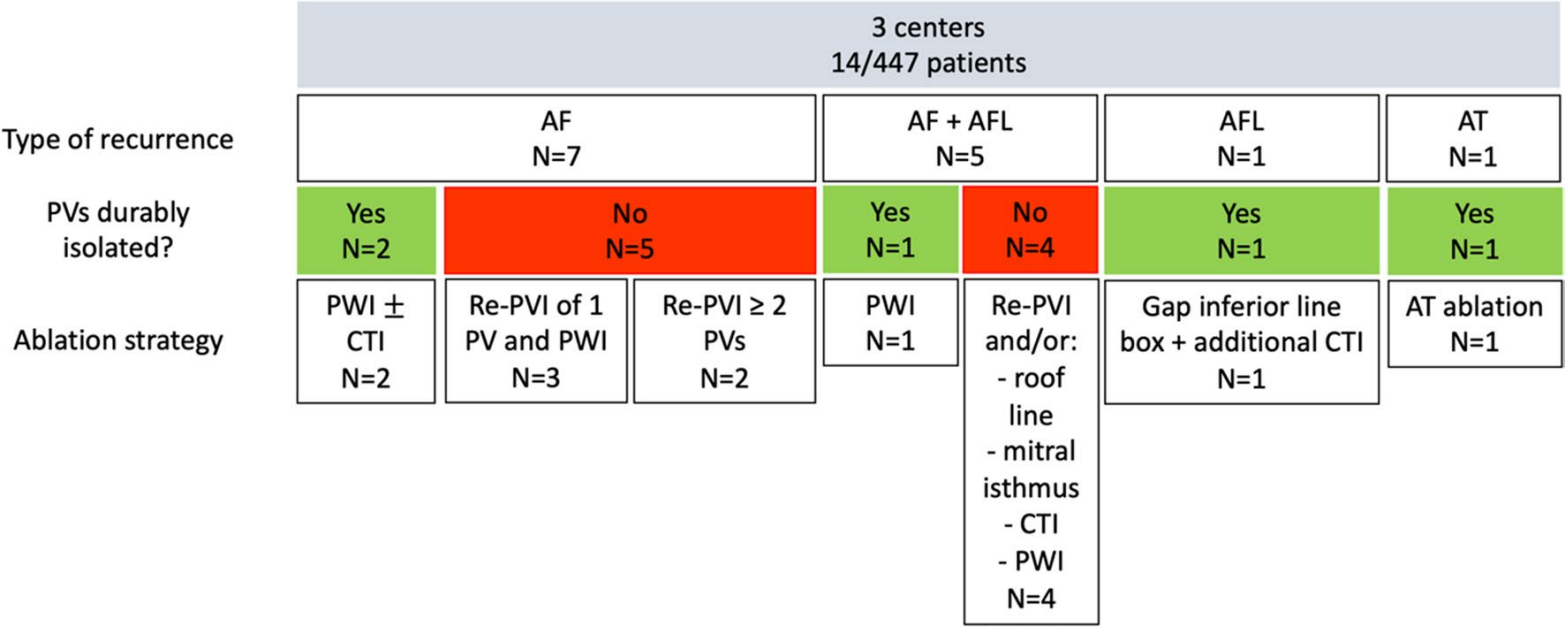
Flow chart

In total, we identified 14 patients (Fig. 1) who had a re-do procedure after an index procedure with PFA. The ratio of re-do procedures to total PFA procedures performed in each centre was 14/447 (3.1%) patients (8/240 (3.3%) in the UMCG, 4/141 (2.8%) in the University Hospital Graz, and 2/66 (3.0%) in the Herz- und Diabeteszentrum NRW). Of these 14 patients, 7 were women and 7 were men (Table 1). Mean age of the group was 61.9±10.8 years, 7 patients suffered from paroxysmal AF, 6 had persistent AF and one patient had long-standing persistent AF. In those in whom a left atrial volume index (LAVI, *n*=10) was available, mean LAVI was 39.4±14.6 ml/m^2^. In 3 out of 14, a left common ostium was encountered while in the remaining 11 patients a normal PV anatomy was found.

**Table 1:** Patient characteristics

	Sex, age	Type AF	LAVI (ml/m^2^), anatomy	BMI (kg/m^2^)	CHA_2_DS_2_-VASc	HT	DM	HF	Stroke	CAD	Baseline AAD use	LVEF (%)
Case 1	F, 74	Paroxysmal	47, LCO	23.9	3	No	No	Yes	No	No	Yes	55
Case 2	M, 64	Paroxysmal	n/a, N	27.4	0	No	No	No	No	No	Yes	58
Case 3	M, 62	Persistent	24, N	32.7	2	Yes	Yes	No	No	No	Yes	n/a
Case 4	F, 52	Persistent	n/a, LCO	29.3	1	No	No	No	No	No	Yes	n/a
Case 5	M, 64	Persistent	43, N	28.1	2	No	No	No	Yes	No	No	50
Case 6	M, 54	Longstanding persistent	44, N	31.6	0	No	No	No	No	No	Yes	55
Case 7	F, 49	Paroxysmal	n/a, N	30.8	2	No	Yes	No	No	No	Yes	n/a
Case 8	F, 74	Paroxysmal	23, N	20.0	4	No	No	No	Yes	No	Yes	58
Case 9	F, 44	Persistent AF	46, N	24.7	2	No	No	No	No	No	Yes	55
Case 10	F, 82	Paroxysmal	40, N	28.9	4	Yes	NO	No	No	No	Yes	60
Case 11	M, 64	Persistent	70, N	26.4	2	Yes	No	No	No	No	No	55
Case 12	M, 70	Paroxysmal	n/a, N	24.9	3	Yes	Yes	No	No	No	Yes	66
Case 13	M, 61	Paroxysmal	21, LCO	25.0	0	No	No	No	No	No	Yes	55
Case 14	F, 53	Persistent	36, N	32.0	2	Yes	No	No	No	No	Yes	55

*F* female, *M* male, *LCO* left common ostium, *N* normal pulmonary vein anatomy, *AF* atrial fibrillation, *AFL* atrial flutter, *n/a* not available

### Recurrences

Mean time to recurrence after index procedure was 5.0±1.8 months. In twelve (85.7%) patients, there was a recurrence of AF and in five of these there was a concomitant recurrence of atrial flutter (3 atypical, 2 CTI-dependent). In the remaining 2 patients, one had a recurrence of (box-dependent) atrial flutter only and the other had a recurrence of atrial tachycardia. In 3 patients, PWI was performed during the index PFA procedure. Mean number of PW applications was 12.0±6.9.

### Re-do procedure

The indication for the redo procedure was recurrent AF (only) in 7/14 patients. 5/14 patients had AF and atrial flutter. In 2/14 patients, only AT/AFL was observed. Patients who had a previous PVI-only PFA procedure all presented with AF. In 5 of these patients, concomitant flutter was observed: in two, this was CTI-dependent and in three, this was an atypical flutter (2 roof-dependent and 1 mitral-isthmus-dependent). Two out of three patients in whom additional PWI was performed during index PFA procedure had recurrent arrhythmia, a box-dependent AFL and an atypical AT (Table 2).

**Table 2:** Procedural details of index PFA procedure

	Type catheter	Skin-to-skin time (min)	Mapping performed?	N^o^ PVs isolated?	Number of applications	Same day discharge?
Case 1	Farawave 35 mm	51	No	4/4 (100%)	32	Yes
Case 2	Farawave 31 mm	Not available	No	4/4 (100%)	32	Yes
Case 3	Farawave 31 mm	59	No	4/4 (100%)	32	Yes
Case 4	Farawave 31 mm	54	No	3/3 (100%)	28 ^†^	Yes
Case 5	Farawave 31 mm	51	No	4/4 (100%)	32	No
Case 6	Farawave 31 mm	78	No	4/4 (100%)	32	No
Case 7	Farawave 31 mm	125	Yes	4/4 (100%)	32	No
Case 8	Farawave 31 mm	89	No	4/4 (100%)	32	No
Case 9	Farawave 31 mm	65	Yes	4/4 (100%)	32 + 8 (PWI)	No
Case 10	Farawave 31 mm	50	Yes	4/4 (100%)	32 + 8 (PWI)	No
Case 11	Farawave 35 mm	91	Yes	4/4 (100%)	32 + 20 (PWI)	Yes
Case 12	Farawave 31 mm	78	No	4/4 (100%)	32	No
Case 13	Farawave 31 mm	49	No	4/4 (100%)	32	Yes
Case 14	Farawave 31 mm	64	No	4/4 (100%)	32	Yes

† 12 total applications were delivered to isolate the left common ostium (+ 16 applications delivered to the right pulmonary veins = 28 applications)

**Fig. 2:**
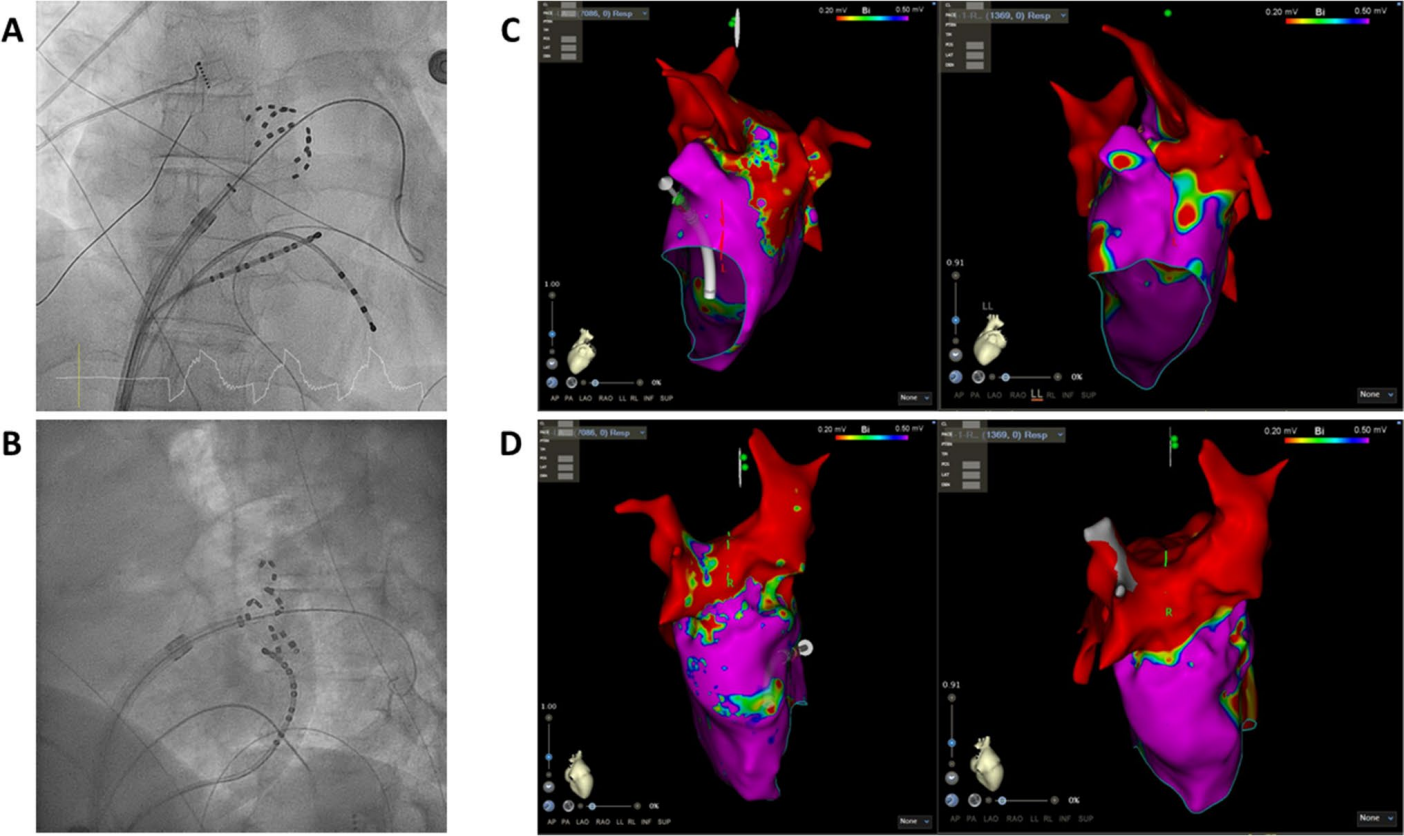
An overview of the index procedure (panel **A** and **B**), of a 3D mapping pre-ablation during the redo (left side panel **C** and **D**) and after radiofrequency ablation (right side panel **C** and **D**). Pulsed field ablation of the left inferior pulmonary vein in basket (panel **A**, AP projection) and flower (panel **B**, LAO 60°) configuration using the Farawave 31-mm catheter, a decapolar catheter in the coronary sinus and a quadripolar catheter in the right ventricle. Panels **C** and **D**: Multielectrode mapping of the left atrium at the repeat procedure revealing gaps in the left carina (panel **C**, left lateral view, before and after ablation), right anterior carina and posterior aspect of the right inferior pulmonary vein (panel **D**, right lateral view, before and after ablation). Voltage thresholds used: 0.2 and 0.5 mV

**Fig. 3:**
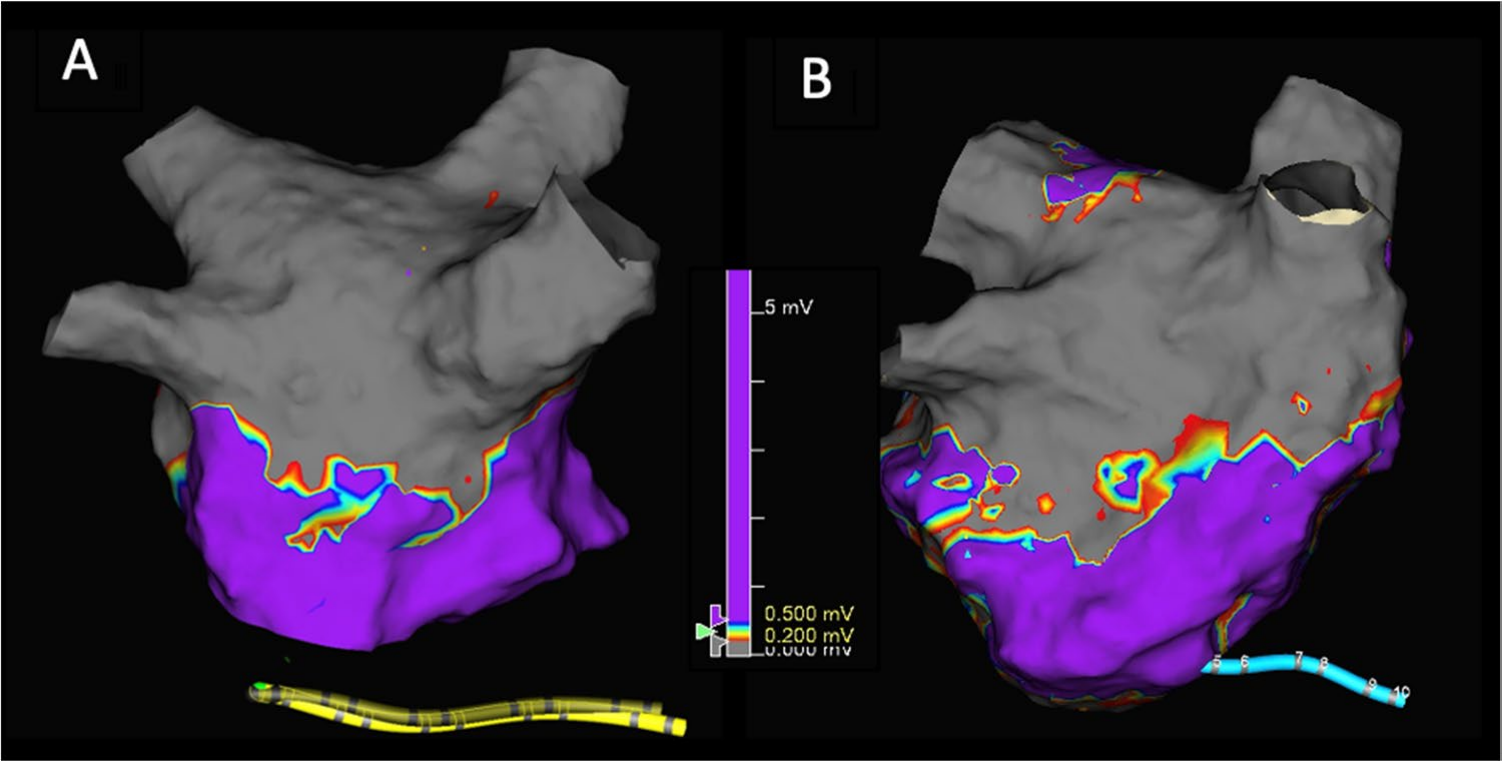
**A** 3-D map of posterior wall isolation performed during index pulsed field ablation; **B** 3-D map during re-do procedure of posterior wall lesion made during index procedure

**Fig. 4:**
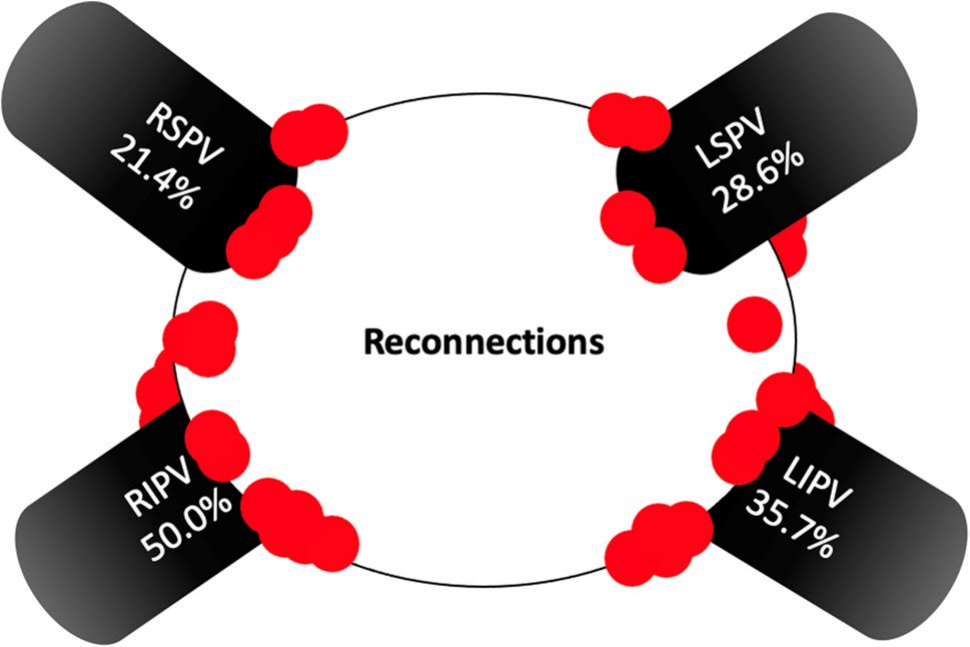
Percentage of reconnection per individual pulmonary vein. In red, the most common location of additional ablation during re-do

**Fig. 5:**
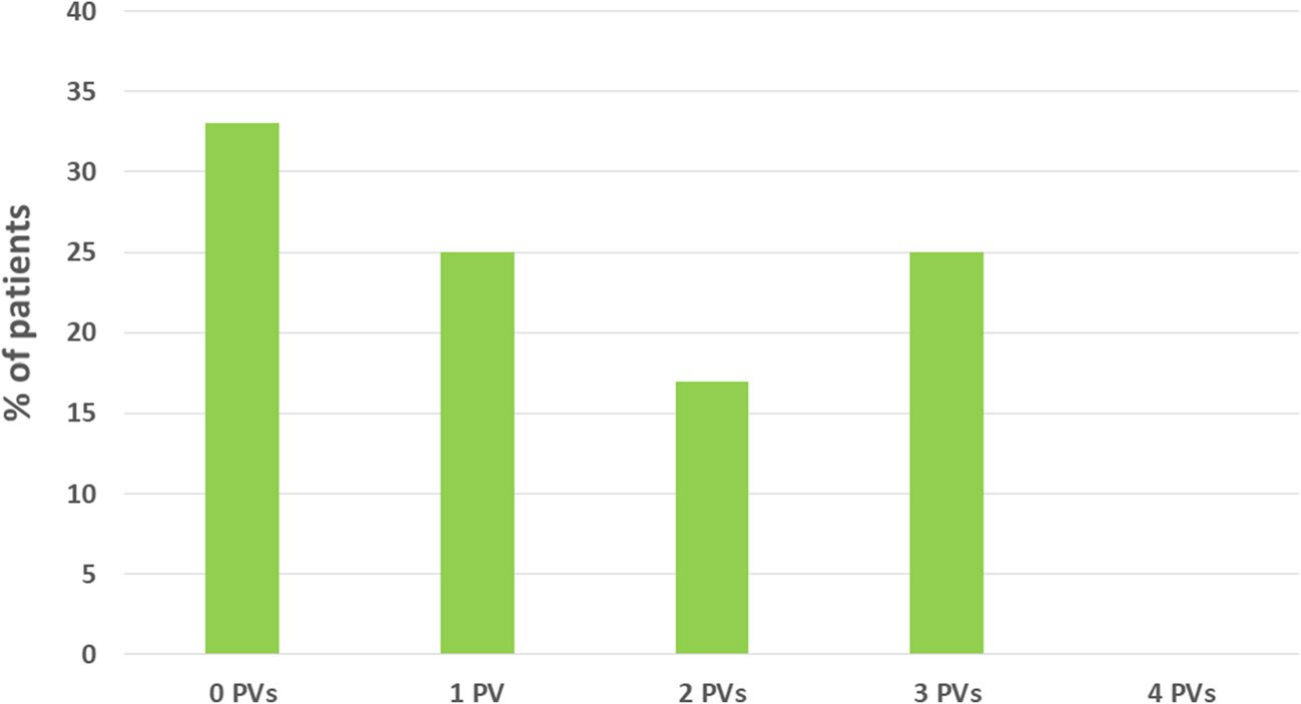
Number of reconnected pulmonary vein at re-do procedure

Mapping of the PVs at re-do demonstrated reconnection in 19/53 (35.8%) of the veins. Remapping of the PW showed incomplete box in 2/3 patients. Figure 2 shows example of reconnection of the PVs. Figure 3 shows example of PWI performed using PFA during index and at redo ablation. Table 3 and Fig. 4 show the rate of PV reconnection per patient. 35.7% of patients had 0 PV reconduction, 21.4% had reconduction in 1 PV, 14.3% in 2 PVs, and 28.6% in 3 PVs. No patient had reconnection of all 4 PVs. Figure 5 shows the rate of reconnection per PV for all patients and site of ablation during redo, from an anterior view. The majority of gaps were found in the posterior-inferior aspect of the RIPV. Figure 3 shows an overview of the index procedure (panel A and B), of a 3D mapping pre-ablation during the redo (left side panel C and D) and after radiofrequency ablation (right side panel C and D).

**Table 3:** Procedural details of the re-do procedure

	Type of recurrence	Time to recurrence? (months)	PV reconnection?	PW reconnection?	Comment	Energy source	Additional ablation
Case 1	AF	6 months	RIPV: posterior/inferior	n/a		RF	PWI
Case 2	AF and AFL	6 months	LSPV: posterior, ridge LIPV: posterior	n/a		RF	Roof, mitral isthmus, and CTI lines
Case 3	AF and AFL	6 months	LSPV: superior, anterior, and posterior LIPV: anterior, inferior, and posterior RSPV: superior and anterior	n/a		RF	Roof and mitral isthmus lines
Case 4	AF	3-6 months	RIPV: inferior and posterior	n/a		RF	PWI
Case 5	AF	3 months	No	n/a		PFA and RF	PWI (PFA) and CTI line (RF)
Case 6	AF	2 weeks	LSPV: anterior to carina LIPV: inferior-anterior RIPV: inferior	n/a		RF	No
Case 7	AF and AFL	5 months	LSPV: ridge and posterior-superior RIPV: posterior-inferior RSPV: anterior-superior	n/a		RF	CTI line
Case 8	AF and AFL	3 months	No. Right carina showed potentials	n/a	Atypical AFL right carina and roof dependent; touch up PW without PWI	RF	PWI
Case 9	AF	6 months	RIPV: anterior inferior	Yes		RF	PWI
Case 10	AT	7 months	No	No	PVs and post Box isolated	RF	Mitral isthmus line
Case 11	AFL	3 months	No	Yes, gap inferior line		RF	PWI and CTI line
Case 12	AF and AFL	7 months	LIPV: posterior-inferior RSPV: anterior-carina RIPV: carina-anterior	n/a		RF	CTI line
Case 13	AF	6 months	LCO: roof LIPV RIPV: posterior	n/a		RF	No
Case 14	AF	6 months	No	n/a		PFA	PWI

*LSPV* left superior pulmonary vein, *LIPV* left inferior pulmonary vein, *RIPV* right inferior pulmonary vein, *RSPV* right superior pulmonary vein, *CTI* cavotricuspid isthmus, *PFA* pulsed field ablation, *PWI* posterior wall isolatio

Ablation strategy beyond reisolating the PVs consisted of mitral isthmus line (3/14), roof line (2/14), posterior box (re) isolation (7/14), CTI line (6/14) (see Table 3) according to substrate involved in the recurrent arrhythmia. No complication occurred during the redo cases.

## Discussion

In this series of 14 patients from 3 centres who had a redo AF ablation following an index PFA ablation, we observed electrical reconnection in 19/53 (35.8%) PVs. In over one-third of patients, all veins were still isolated. In none of the patients, reconnection of all veins occurred. During re-do ablation, isolation of the posterior wall was confirmed in 1/3 patients who had posterior wall ablation during the index procedure. The predominant recurrent arrhythmia following PFA PVI was AF (85.7%), but 35.7% of patients had concomitant AFL recurrence. Two out of three patients in whom PFA PWI was performed had AT/AFL only as the recurrent arrhythmia.

### PV lesion durability after PFA AF ablation

PVI remains the hallmark of any AF ablation strategy and has been proven feasible with all current techniques [1]. In patients with recurrence of AF, most have evidence of PV reconnection [1]. Reported rates of PV reconnection following PVI with cryoballoon or radiofrequency ablation range from ∼22–38% to as high as 62,5% (Fig. 4) [9]. There is a need for an energy source that can create transmural lesions and durable PVI without collateral damage. PFA appears to be a promising candidate, as shown by findings from early clinical investigations [4].

The PEFCAT and IMPULSE trials reported on the PV reconnection rate in patients that had previous PFA procedures during scheduled remapping 93.0±30.1 days after the index procedure [7]. They demonstrated durable PVI in 84.8% of PVs (64.5% of patients), and 96.0% of PVs (84.1%), who were treated with biphasic energy PFA platform. Of note, mapping was performed irrespective of AF recurrence. Conversely, we present the first data on PV reconnection rates in patients who had documented recurrence of AF or AFL/AT and were referred for redo procedure. We report durable PVI in 61% of the PVs. This is lower than 96% reported in previous studies [3, 7]. This may be explained by the fact that we only remapped patients who had AF/AFL recurrence. It has been demonstrated before that reconnection rates are higher in patients with AF recurrence compared to patient without [10].

PFA may offer a highly selective nature of its lesion formation, promising more durable and transmural PVI and resultant lower recurrence rates. PersAFOne is a single-arm study that evaluated bipolar, biphasic PFA utilizing a pentaspline catheter for PVI and left atrial posterior wall (LAPW) ablation in 25 patients with persistent AF [3]. Invasive remapping was performed after 2–3 months to assess lesion durability in these patients. Durable isolation was demonstrated in 82 of 85 PVs (96%) and 21 of 21 LAPWs (100%). Furthermore, the recently published MANIFEST-PF study showed that PFA was efficacious for PVI and expressed a safety profile consistent with preferential tissue ablation [4]. However, the frequency of catheter complications (tamponade, stroke) underscores the need for improvement. In our case series, we observed durable PVI in 35.7% and durable PWI in 33.3% of patients, and we registered no minor or major complications during the index or re-do procedures.

Recently, Kueffer et al. reported on the recurrence rate of AF after PVI using PFA in 41 patients [11]. Recurrence of AF was assessed by seven-day Holter ECGs performed at 3 and 6 months after ablation and was documented in 5 patients (12%), 1 (6%) paroxysmal AF and 4 (17%) persistent AF, respectively. Redo procedures were performed in three patients and showed 100% durable isolation without conduction breakthrough in all PVs and no evidence of lesion regression. In this study, we illustrate that PV reconnection can take place after acute PV isolation and similarly to the results found in the Fire and ICE study, we showed that different degrees of PV reconnection can occur [8]. In our series, PV reconnections following PFA appear to be largely located at the right carina, anterior aspect of the RSPV, posterior-inferior aspect of the RIPV, posterior aspect of the LSPV, and posterior-inferior aspect of the LIPV. This pattern is similar to that commonly found after thermal ablation [9]. This may be associated with the greater thickness of the myocardium in these areas and limited catheter manoeuvrability (RIPV). Others have also shown that the level of PVI, i.e. quantification of the ablated area, was not different when comparing PFA, radiofrequency and cryoballoon — a finding comparable with ours in the current series [12]. Several reasons might explain why the PVs were not durably isolated. Contact is likely an important issue and is shown to be optimized by using intracardiac echo, increasing the durability of PVI (98.2% versus 91.8%) [7]. Although it is still a 2D image of a 3D multispline cathether. Also, when adopting a new technique, there might be a learning curve influencing optimal contact and success of the procedure [13]. Furthermore, specific patient characteristics with regard where reconnection occur may also further reduce the chance of long term success of the procedure and should be further investigated [14–16]. Even though PFA dosage has been thoroughly investigated by pre-clinical studies, studies investigating where additional applications may lead to more durable isolation are warranted.

### Type of recurrence after PFA

In this small series, we describe the type of arrhythmia recurrence following PFA ablation. The majority of patients had recurrence of AF (85.7%). This is comparable to what has also been described for other ablation energy sources [8]. We also observed cases of (concomitant) atrial flutter for which different lesion sets were employed. Interestingly in patients who had a previous PW ablation, 2/3 had recurrence of AT/Aflutter. These were patients who had PWI for ablation of persistent AF. In one of the patients the atrial flutter was a result of an incomplete box, and the re-entrant circuit included the PW. At this point in time, it is too early to suggest this as a common finding after PFA ablation of the PVs and the PW.

### Strengths, limitations, and future studies

To date, very few studies have reported on the experience in clinical practice with PFA for treatment of AF, and even fewer have reported the findings at redo procedures after PFA. Therefore, this study is one of the first to provide insights into the durability of PFA lesions evaluated during re-do ablation. Due to the very recent adoption of this novel ablation modality in all three centres involved, very few patients had undergone a redo procedure at the time of this study, resulting in a small sample size. For the same reason, no follow-up was available to further assess the efficacy of redo ablation after PFA. As this was not a clinical trial, follow-up was part of routine outpatient clinic visits. The criteria for proposing the redo ablation could therefore be different amongst the centers. In addition, variability in protocol and experience between centres and operators may have limited the strength of our results, although it has been shown that outcomes are comparable between different operators of different levels of experience [17]. In addition, all three centres recently started with PFA and a potential learning curve may have had an impact on our results. It is likely that with more experience, reconnection rates will go down even more. Future studies investigating findings at redo ablation following PFA in a larger patient cohort are warranted to confirm and build up on the findings of our study. More extensive follow-up after redo would also allow to better understand the role of PFA in the ablation treatment (including durability of PVI and PWI) strategy of AF.

## Conclusion

In conclusion, we observed durable PV isolation in 64.2% of the PVs in patients with recurrence of AF/AFL after PFA PVI. In over one-third of patients, all PVs were isolated. The predominant recurrence arrhythmia following PFA PVI-only procedures was AF. Future studies are warranted to further understand the long-term effects of PFA.
