# Correction: Electrophysiological findings during re-do procedures after single-shot pulmonary vein isolation for atrial fibrillation with pulsed field ablation

**DOI:** 10.1007/s10840-023-01610-z

**Published:** 2023-08-04

**Authors:** Federico Tancredi Magni, Daniel Scherr, Martin Manninger, Christian Sohns, Philip Sommer, Tatevik Hovakimyan, Yuri Blaauw, Bart A. Mulder

**Affiliations:** 1 Department of Cardiology, University of Groningen, University Medical Center Groningen, P.O. Box 30.001, 9700, RB Groningen, The Netherlands; 2 Department of Medicine, Division of Cardiology, Medical University of Graz, Graz, Austria; 3 Clinic for Electrophysiology, Herz- und Diabeteszentrum NRW, Bad Oeynhausen, Germany; 4 Department of Cardiac Arrhythmology, Nork-Marash Medical Center, Armenak Armenakyan 108/4, Yerevan, Armenia

**Correction to: Journal of Interventional Cardiac Electrophysiology**

10.1007/s10840-023-01559-z

Figure 5 in the original version of this article has been replaced with the figure as shown below.

**Fig. 5:**
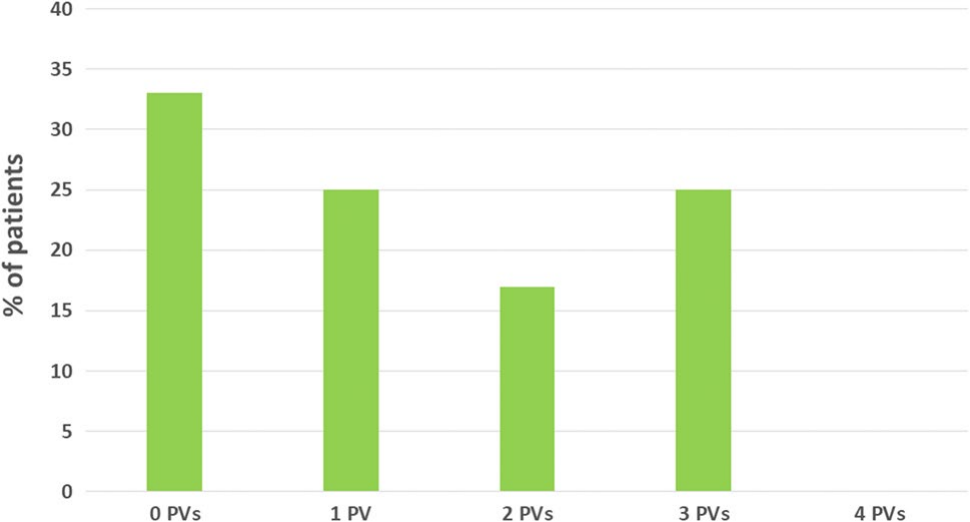
Number of reconnected pulmonary vein at re-do procedure

The original article has been corrected.

